# Copper-based water repellent and antibacterial coatings by aerosol assisted chemical vapour deposition†
†Electronic supplementary information (ESI) available. See DOI: 10.1039/c6sc01150k


**DOI:** 10.1039/c6sc01150k

**Published:** 2016-04-20

**Authors:** Ekrem Ozkan, Colin C. Crick, Alaric Taylor, Elaine Allan, Ivan P. Parkin

**Affiliations:** a Materials Chemistry Research Centre , Department of Chemistry , University College London , 20 Gordon St , London , WC1H 0AJ , UK . Email: i.p.parkin@ucl.ac.uk ; Tel: +44 (0)207 679 4669; b Department of Chemistry , Imperial College London , South Kensington Campus , London , SW7 2AZ , UK; c Department of Electronic and Electrical Engineering , University College London , Torrington Place , London , WC1E 7JE , UK; d Division of Microbial Diseases , UCL Eastman Dental Institute , University College London , 256 Grays Inn Road , London , WC1X 8LD , UK

## Abstract

The adhesion and proliferation of bacteria on solid surfaces presents a major challenge in both healthcare and industrial applications. In response to this problem, an effective and simple method is reported to fabricate superhydrophobic antibacterial copper coated polymer films *via* aerosol assisted chemical vapor deposition (AACVD). The material is characterized using a range of techniques including electron microscopy, water contact angle measurement and elemental mapping. The antibacterial activity of the modified film is tested against the Gram-negative bacterium, *Escherichia coli*, and the Gram-positive bacterium, *Staphylococcus aureus* and the film shows highly significant antibacterial activity against both bacteria (>4 log reduction in bacterial numbers) in 15 min and 60 min, respectively. In addition, all the CVD modified samples results in a significant reduction in bacterial cell adhesion compared to the control materials. Thus, we report a new film type that has dual mode of action-the superhydrophobicity helps limit cell adhesion combined with a cytotoxic copper induced bacteria kill.

## Abstract 2

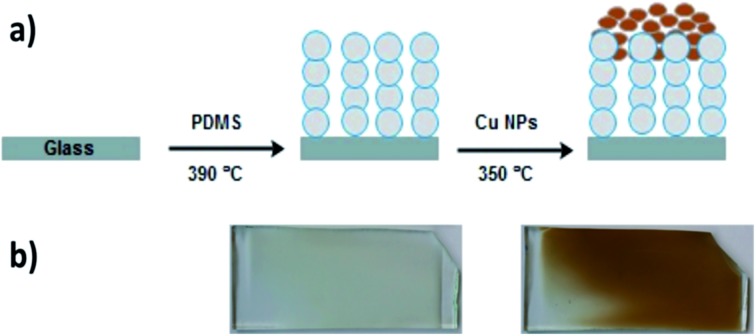
The adhesion and proliferation of bacteria on solid surfaces presents a major challenge in both healthcare and industrial applications.

## 1 Introduction

Although nano-biotechnology and nanofabrication methods have developed remarkably over recent years, there is still an urgent need to design and generate new antimicrobial surfaces.^1^ Microorganisms have evolved a multitude of adaptive mechanisms in order to colonize surfaces.^2^ Bacterial colonization of surfaces is known to have an adverse effect on the function of a diversity of materials such as textiles, medical catheters, dental implants and resistant contact lenses.^3^–^5^ For example, hospital-acquired infections, many of which arise from catheter colonization, cost over £1 billion to the United Kingdom economy per year^6^ with 300 000 cases resulting in approximately 5000 deaths per year.^7^

Antibacterial surfaces may either (i) prevent the attachment of bacteria *i.e.* anti-biofouling activity or (ii) disable bacteria that do adhere to the surface *i.e.* bactericidal activity.^8^,^9^ One promising approach to prevent biofouling is to employ superhydrophobic surfaces. Such surfaces show excellent non-wetting properties due to a water angle greater than 150° and water droplets easily roll-off carrying away dirt particles and bacteria.^10^ Several studies have shown that surface wettability has a major effect on bacterial adhesion. For example, Arima *et al.* demonstrated that bacteria could effectively adhere onto surfaces with contact angles of 40–70°.^11^ On the other hand, Freschauf *et al.* found that different shrink-induced superhydrophobic polymers significantly prevented the adhesion of *E. coli*.^12^ However, while antibiofouling surfaces can reduce bacterial adhesion, they are not able to inactivate adherent bacteria. Metal nanoparticles (NPS) such as copper can effectively kill bacteria,^13^ we predicted that a bioactive surface with both antibiofouling and bactericidal properties would be most effective at reducing the load of colonizing bacteria relative to materials displaying either property alone.

Antibacterial NPs comprise metals (*e.g.* silver) and metal oxides (*e.g.* zinc oxide and copper oxide), naturally-occurring antibacterial substances (*e.g.* enzymes), carbon-based materials, and surfactant-based nanoemulsions.^14^–^16^ It is believed that high surface area to volume ratios and novel chemico-physical features of different nanomaterials are associated with efficient antimicrobial activities.^17^ Known antimicrobial mechanisms of nanomaterials include: (i) photocatalytic generation of reactive oxygen species (ROS) that lead to damage of bacterial and viral components, (ii) compromising of the bacterial cell wall or membrane, and (iii) interruption of energy transduction within bacterial membranes.^18^

Different nanomaterials including TiO_2_, ZnO and SiO_2_ have been shown to possess potent antibacterial properties. Among these metal-based NPs, copper-based nanomaterials have attracted considerable attention because of their high redox potential and relatively low-cost of production.^19^ Copper is also relatively non-toxic to mammals,^20^ but shows strong toxicity against a broad range of microorganisms.^21^ This can form the basis of a new approach for antibacterial treatment. Copper has been known for some time to be an excellent biocide, and both copper ions and NPs have shown antibacterial activity against a broad range of bacterial species.^22^–^24^ Recently, Hassan *et al.* reported that both copper and copper-based films showed excellent antibacterial activity against both Gram-positive and Gram-negative bacteria.^25^

Herein, we report a technique for coating small-sized copper nanoparticles (Cu NPs) onto a curable silicone polymer, polydimethylsiloxane (PDMS), facilitated by a simple two-step deposition process using aerosol assisted chemical vapour deposition (AACVD). To our knowledge this coating is unique in displaying two functionalities; superhydrophobicity preventing bacterial adhesion and a potent antibacterial effect from the Cu-NPs.

## 2 Results and discussion

### 2.1 Materials synthesis and characterization

**Fig. 1:**
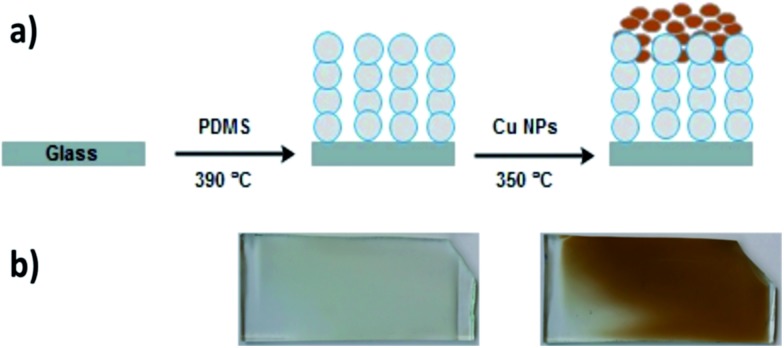
(a) Schematic to show fabrication of Cu NPs coated PDMS. (b) Images of Sylgard 184 polymer deposited *via* AACVD at 390 °C – on the left – and after the NP deposition at 350 °C – on the right. The sample dimensions are 14 cm × 4.5 cm × 0.5 cm.

Nanoparticle incorporated polydimethylsiloxane (PDMS) was prepared *via* a facile two-step AACVD process. Firstly, AACVD was carried out using a chloroform solution of the thermosetting Slygard-184 (base and curing agent) at 390 °C, where the chloroform solvent evaporates and the thermoset polymer is cured. This followed by a methanol solution of Cu NPs, which was deposited onto the as-prepared polymer matrix at 350 °C by a second CVD step (Fig. 1a). While PDMS films are white-opaque, copper-containing films are metallic brown in colour (Fig. 1b). The films were uniformly deposited onto glass substrates.

The presence of the Cu-NPs onto the superhydrophobic polymer film was confirmed by UV-vis spectroscopy, scanning electron microscopy (SEM), transmission electron microscopy (TEM), energy dispersive X-ray spectroscopy (EDX) and functional testing. UV-vis spectra of polymer samples were measured between 250 nm and 4000 nm but a small section was isolated (see ESI, Fig. S1†). While pure PDMS does not have any absorbance in the UV region, after coating by NPs, a broad peak centered at 310 nm was observed, which is characteristic of Cu nanocrystals.^26^

**Fig. 2:**
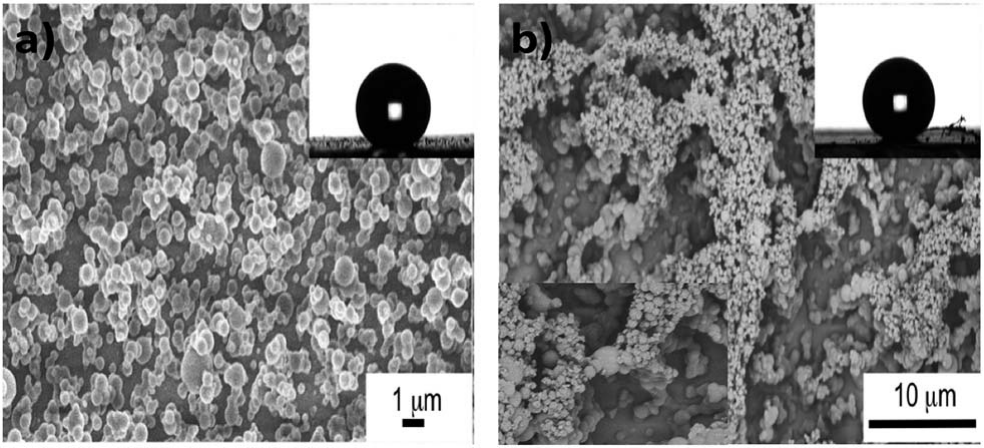
SEM images of (a) PDMS film grown by AACVD of Slygard 184 in chloroform at 390 °C and (b) after coating with Cu NPs at 350 °C (respective inset (bottom left) shows the higher magnification image (1 μm)). Inset: images of water droplets in contact with the prepared polymer surfaces.

The morphology of the films was investigated using scanning electron microscopy (SEM) and transmission electron microscopy (TEM). Cu NPs were deposited onto PDMS matrix through the AACVD deposition process. Therefore, they were expected to be anchored on the PDMS surface and this was confirmed by both SEM and TEM images. SEM images revealed that PDMS films by AACVD had a very rough surface consisting of interlocking particles with surface protrusions around 3–5 μm in length and spherical Cu NPs were uniformly attached to the surface protrusions (Fig. 2).

**Fig. 3:**
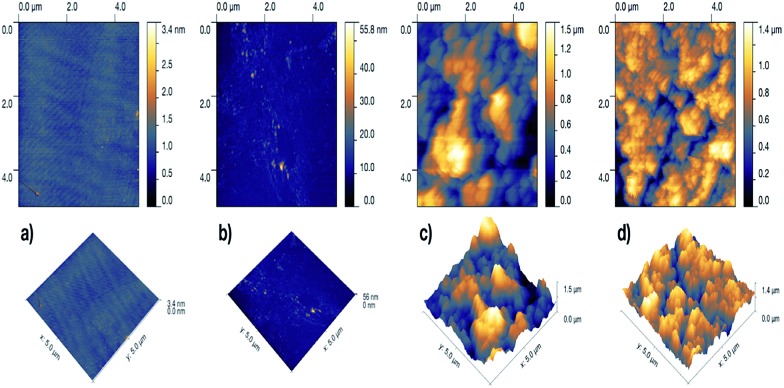
Two-dimensional (top) and three-dimensional AFM images of (a) plain glass, (b) plain PDMS, (c) CVD-treated PDMS and (d) Cu-coated CVD-treated PDMS.

The surface morphology and roughness of the samples was further examined by atomic force microscopy (AFM). The two-dimensional and three-dimensional AFM images of the samples are shown in Fig. 3. Both the glass and the PDMS sample had rather smooth surface with *R*_rms_ (root-mean-square-roughness) of 0.132 nm and 2.175 nm, respectively. After CVD treatment with PDMS, the glass surface dramatically roughened with *R*_rms_ of 0.278 μm. However, with Cu-incorporation, the roughness of the hybrid film slightly reduced to *R*_rms_ of 0.230 μm.

The surface of bare glass was hydrophilic with a water contact angle of 48 ± 3° while bare PDMS was hydrophobic itself with a water contact angle of 111 ± 5°. During the AACVD process, PDMS with low-surface energy was cured resulting in a highly rough surface with water contact angle up to 155 ± 2° (inset of Fig. 2a). No significant change in the wetting properties was observed upon coating of the Cu-NPs into the polymer matrix with a contact angle of 151 ± 2° (inset of Fig. 2b). While the water droplets (10 μl) adhered to the S-PDMS surface slide away at a tilting angle of less than 10°, the Cu-PDMS surface is extremely slippery compared to the S-PDMS, enabling the water droplets to readily roll off, even at 1° tilt angle. Water bouncing has previously been used as a versatile measurement of superhydrophobicity, whereby the number of bounces on a surface is directly correlated with the material's ability to repel water.^29^ A water-repelling test was performed on the samples at the impact velocity of 1.2 ms^–1^ from a height of 7.5 cm (see ESI, Movie S1†). The movie shows that the water droplet makes good contact on both the glass and the PDMS sample. On the other hand, the water droplet impacts the superhydrophobic surfaces and bounced up without wetting them.

**Fig. 4:**
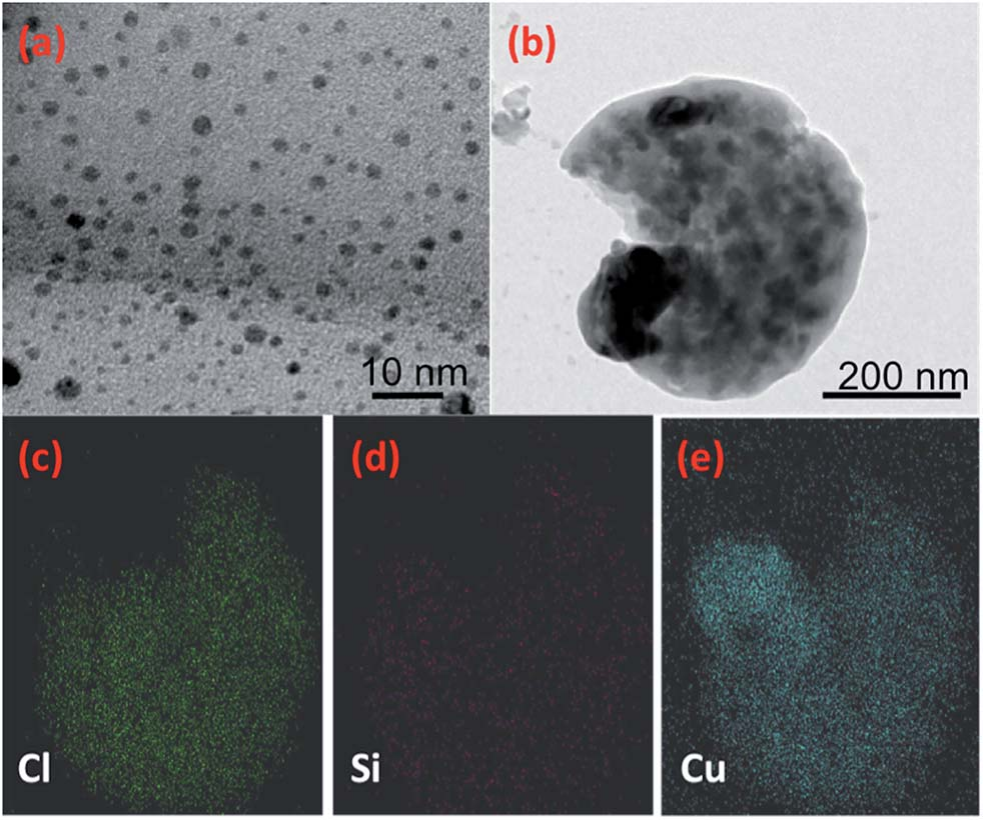
TEM images (a) colloidal Cu NPs and (b) Cu-PDMS composite and (c–e) energy dispersive X-ray spectrum and mapping of the Cu-PDMS composite for the elements Cl, Si and Cu.

A simple green route was utilized to produce the Cu NPs by drop-wise addition of l-ascorbic acid to copper(ii) chloride solution at 70 °C. Further heating resulted in a dark brown solution indicating that reaction was completed. Non-toxic antioxidant, l-ascorbic acid, was used as both reducing Cu^2+^ to Cu^0^ and capping the NPs to prevent aggregation and oxidation.^27^ TEM images of the particles reveal that the Cu NPs are well-separated, spherical without any agglomeration (Fig. 4a and b). The average particle size was determined to be 3.56 ± 0.8 nm (see ESI, Fig. S3a†). Fig. 4c–e shows the TEM image of the Cu-PDMS composite and its corresponding elemental mapping. It can be clearly seen that Cu NPs were distributed throughout the polymer matrix. Also, elemental distributions from the EDS spectrum indicate that the elements Cu, Si and Cl are the main constituents (see ESI, Fig. S3b†). While the elements Cl and Cu are associated with the synthesis of the NPs, the Si comes from the polymer itself consisting of a flexible (Si–O) backbone and a repeating (Si(CH_3_)_2_) unit.^28^

### 2.2 Microbiological testing

The antibacterial activities of the following samples under dark conditions were evaluated against two model hospital-associated pathogens; the Gram-negative bacterium, *E. coli*, and the Gram-positive bacterium, *S. aureus*; a glass sample (control), a bare polymer sample (PDMS), a CVD treated polymer (S-PDMS) and a Cu-coated CVD-treated polymer sample (Cu-PDMS).

**Fig. 5:**
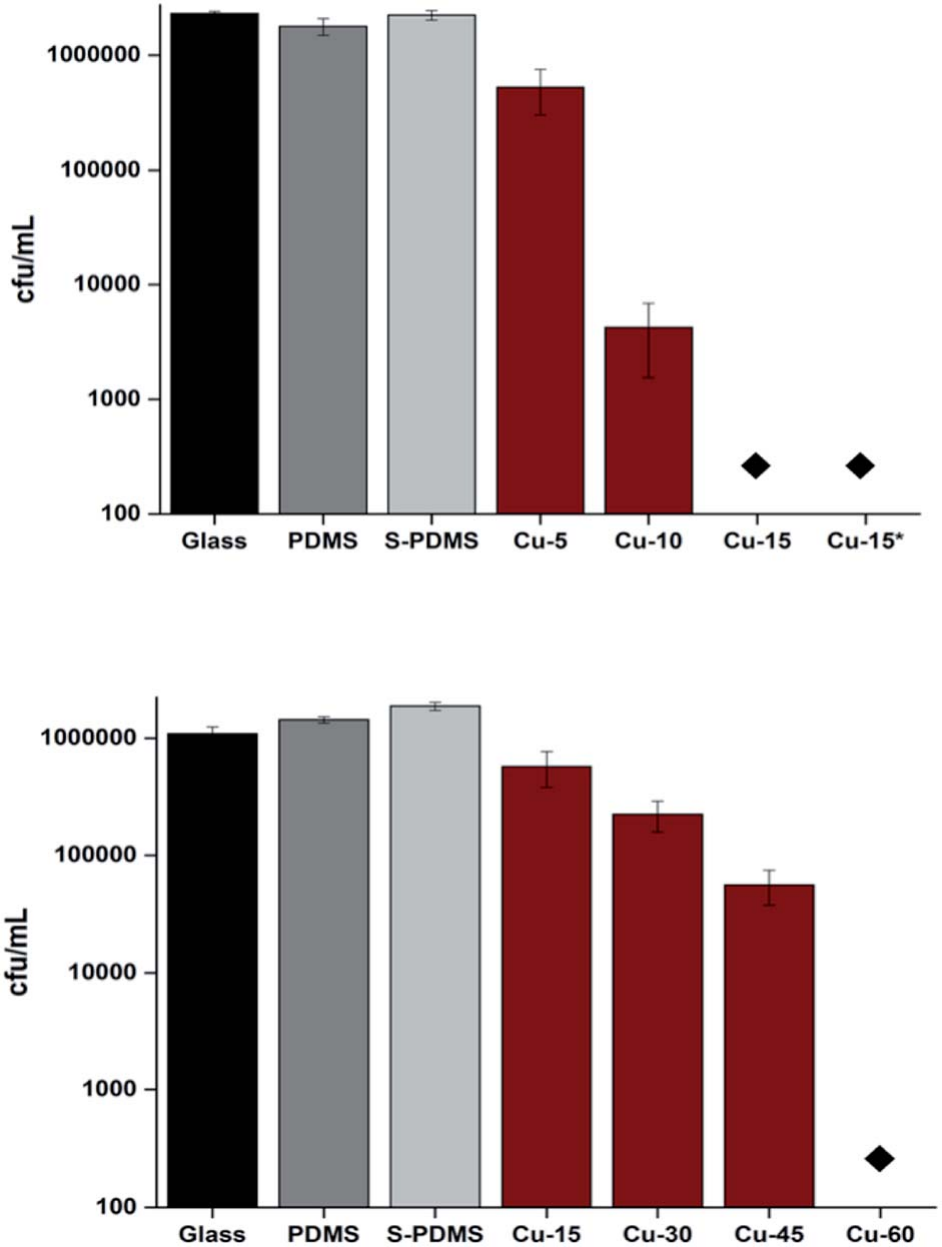
Numbers of *E. coli* after incubation on the surfaces of the samples in the dark (top) and numbers of *S. aureus* (bottom) after incubation on the surfaces of the samples in the dark. The asterisks indicate where the bacterial numbers are below the detection limit of 100 cfu ml^–1^. Black bars refer to uncoated glass. Gray bars refer to bare PDMS (no nanoparticle). White–gray bars refer to CVD treated PDMS (S-PDMS). Brown bars refer to Cu NPs coated CVD-PDMS (Cu) with different time intervals. Bare glass, PDMS and S-PDMS samples were exposed to *E. coli* and *S. aureus* for 15 min and 60 min, respectively while dip-coated Cu-PDMS (Cu*) was exposed to *E. coli* for 15 min.

Fig. 5 shows that no reduction in the numbers of bacteria was observed on the samples after 15 min compared to the control sample when Cu NPS were absent. However, all Cu-coated samples achieved significant bacterial kill for all exposure times (*P* < 0.01). A 2.3-log reduction in bacterial numbers was achieved after 10 min of exposure to the sample coated with Cu NPs and >4 log reduction was achieved after 15 min with the materials containing Cu NPs.

In the case of *S. aureus*, there was no detectable kill of *S. aureus* on the surface of either bare PDMS or CVD treated PDMS after 1 h in the dark compared to the glass sample. However, the samples coated by Cu NPs demonstrated a 0.69 log reduction in viable bacteria after 15 min (*P* < 0.01). Moreover, a 1.4-log reduction in viable bacteria was observed after 45 min on the surface of the Cu coated PDMS samples whereas after 1 h the number of *S. aureus* was reduced to below the detection limit (>4 log reduction; *P* = 0.02).

The antibacterial mechanism of Cu NPs is not yet fully understood,^30^ however several mechanisms have been suggested. Some studies propose that copper increases intracellular ROS production causing oxidative stress and DNA damage.^31^,^32^ Furthermore, copper may damage the cell membrane.^33^ Moreover, their antibacterial activity may be attributed to the continuous release of copper ions under wet conditions^34^ that attach to the bacterial cell wall. This interaction may lead to cell death by disrupting the cell membrane.

### 2.3 Anti-adhesion properties

**Fig. 6:**
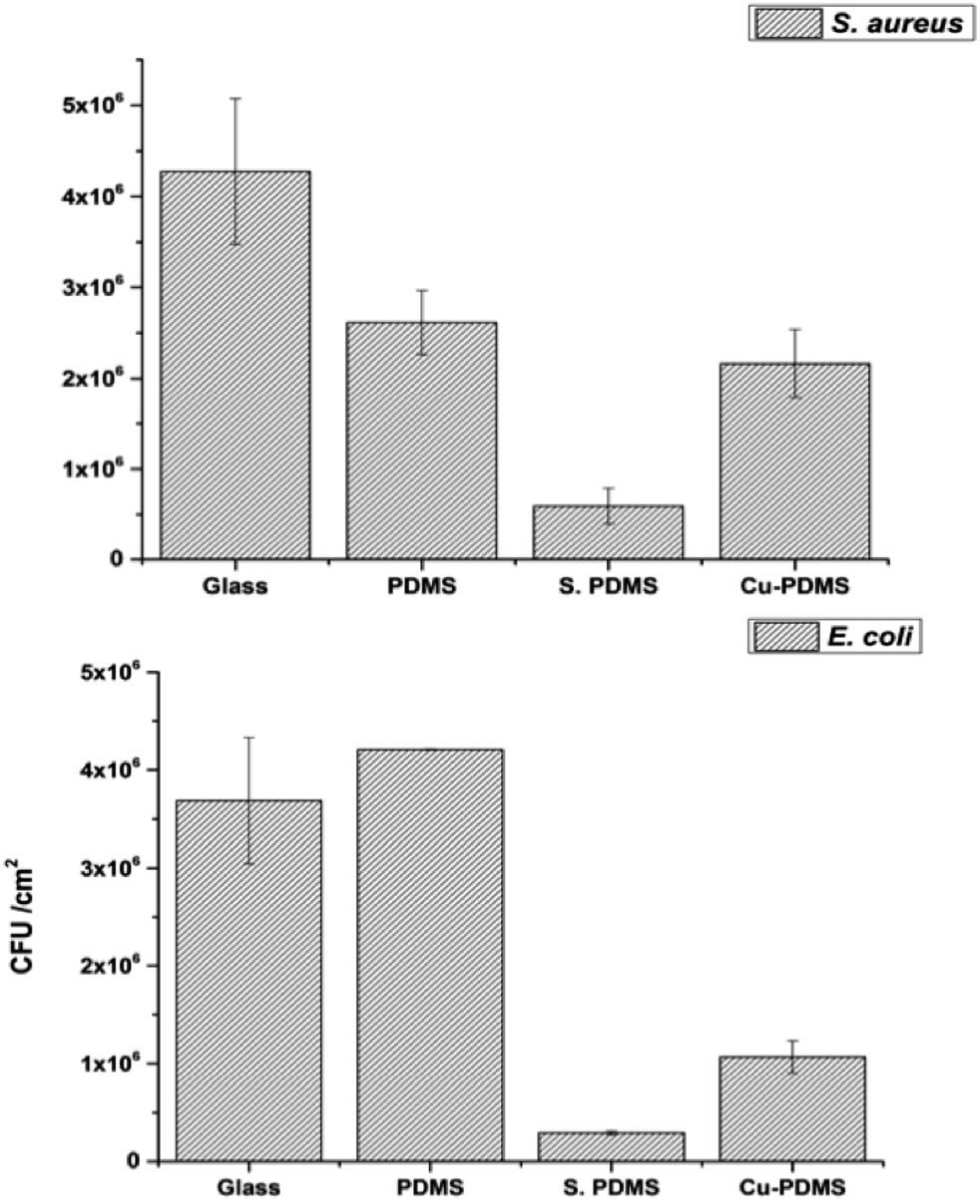
Adherence of *S. aureus* (top) and *E. coli* (bottom) to the sample surfaces including uncoated glass (glass), bare PDMS (PDMS), CVD treated PDMS (S-PDMS) and Cu NPs coated CVD-PDMS (Cu-PDMS).

A bacterial adhesion test was carried out with app. 10^7^ cfu ml^–1^ of the two selected strains after 1 h period of incubation. It was observed that bacterial adhesion rates on the samples were greatly affected by surface wettabilities as shown in Fig. 6. The glass sample had the greatest attachment of *S. aureus*. With high contact angles the number of adherent bacteria was significantly reduced compared to glass alone with 38% and 80% of the bacteria adhering to the bare PDMS and Cu-PDMS, respectively. CVD treated PDMS was superior to Cu-PDMS inhibiting 87% of bacterial attachment compared to glass alone.

With *E. coli*, the highest level of adherence was observed on bare PDMS while the S-PDMS showed the least attachment (Fig. 6). The number of bacteria adhered to the Cu-PDMS surface was 50% of that on the glass surface. These results demonstrated that CVD modification improves the non-fouling nature of the bare PDMS surfaces against *E. coli* and *S. aureus*.

The small surface features that make up most superhydrophobic materials means that most have a poor mechanical durability; this is the main problem limiting the worldwide application of these coatings. Therefore, robustness of the superhydrophobic samples was tested using a standardized scotch tape test (ASTM).^35^ While CVD treated PDMS passed the test, the Cu NPs coating were removed from the substrate that was used in the antibacterial test because it had weak adhesion strength with the polymer. To overcome this problem, the Cu coated polymer sample was dip-coated into a %1 PDMS-CHCl_3_ solution (withdrawal rate of 120 cm min^–1^) followed by curing so that a thin layer of PDMS was deposited into the micropillars. Then, the dip-coated Cu-PDMS before and after the test was examined with SEM images, contact angle measurements and antibacterial test. The dip-coated Cu-PDMS was again tested against *E. coli* and it reached the detection limit (>4 log reduction) in 15 min (Fig. 4a). Also, the images confirmed that the coating remained stable that retain superhydrophobicity (see ESI, Fig. S4†).

PDMS has been used in a wide range of applications including sensors and microfluidic channels because of its properties including high flexibility, low cost, non-toxic nature, chemical inertness and easy preparation.^36^–^38^ Therefore we anticipate that the loading of Cu NPs onto PDMS may extend its use in antibacterial applications from air filters to touch surfaces such as door handles and bed rails in clinical environments. Consequently, this may help disrupt the cycle of transmission of microorganisms between patients, healthcare staff and the environment.

## 3 Conclusions

In this work, we have successfully fabricated a novel superhydrophobic antibacterial surface by combining PDMS and Cu-NPs *via* AACVD. The modified sample showed superhydrophobicity with a water contact angle of 151° as well as remarkable water bouncing properties. It was potent at killing suspensions of *S. aureus* in just 1 hour and *E. coli* in just 15 min, with a minimum of a 4-log reduction (99.99 kill) in the numbers of both bacteria. Moreover, the CVD treated samples greatly prevented the adhesion of both types of bacteria compared to glass and PDMS samples. This novel hybrid surface may help decreasing the incidence of infection in healthcare environments as it works in a novel two fold manner-preventing bacteria sticking and inactivating those that do.

## 4 Experimental

### 4.1 Materials

CuCl_2_·2H_2_O (Sigma, UK) and l-ascorbic acid (Sigma, UK) were utilized in nanoparticle synthesis. Sylgard® 184 Silicone Elastomer was purchased from Dow Corning Corporation Ltd, which consists of two part silicone elastomers (base and curing agent). The precursor, Sylgard 184, can be cross-linked with the curing agent and the final polymer is polydimethylsiloxane (PDMS). Laboratory solvents were purchased from Fisher Scientific Limited and of the highest possible grade.

### 4.2 Preparation of AACVD precursors and the films

#### 4.2.1 Polymer solution

The two components of Sylgard® 184 Silicone Elastomer (0.70 g) were dissolved in (70 ml) with rapid stirring. To prevent premature curing the mixture was used immediately after stirring for deposition studies.

#### 4.2.2 Nanoparticle solution

Cu nanoparticles were prepared according to Xiong *et al.* 5 ml of the solution was diluted to 50 ml with methanol.^27^

#### 4.2.3 AACVD deposition

The depositions were carried out in a cold-walled horizontal-bed CVD reactor. The reactor contained top and bottom plates, both composed of SiO_2_ coated barrier glass (dimensions: 140 × 45 × 5 mm; barrier thickness 50 nm) supplied by Pilkington NSG. A carbon block on which the bottom plate was placed heated the CVD reactor. The top plate was positioned 8 mm above and parallel to the bottom plate, the complete assembly was enclosed within a quartz tube. The aerosol of the precursor solution was generated using a PIFCOHEALTH ultrasonic humidifier with an operating frequency of 40 kHz and 25 W of power. The aerosol generated was moved to the reactor using a nitrogen gas flow *via* PTFE (polytetrafluoroethylene) and glass tubing, where it entered between the top and bottom plates. The reactor waste gas left *via* an exhaust.

Nitrogen flow carried the vapour from the flask until all liquid was gone, which took typically 50–60 min per deposition. The depositions were carried out at 390 °C and 350 °C respectively with an air flow of 0.8 l min^–1^. The thin films of superhydrophobic PDMS and Cu NPs on PDMS were deposited. Then, the heated carbon block was then turned off and allowed to cool to room temperature; the nitrogen flow was left on for a further 15 min. The cooled plates were removed and handled in air. The deposition of the films occurred to the top plate.

### 4.3 Characterization techniques

A Perkin-Elmer Lambda 950 UV-vis Spectrometer was used to measure the UV-vis absorption spectra analyses of the polymers within the range 250–400 nm. Water droplet contact angles were measured using a First Ten angstroms 1000 device with a side mounted rapid fire camera fire casting ≈3 water droplet on the surface of each sample and 5 replicates on fresh samples were performed. Scanning electron microscopy was performed using secondary electron imaging on a JEOL 6301 field emission instrument with Oxford instruments EDX spectrometer attached. Transmission electron microscopy (TEM) images were recorded using a JEOL JEM 1200EX with a 4 megapixel Gatan Orius SC200 charge-coupled device (CCD) camera at an acceleration coltage of 120 kV. Powder X-Ray Diffraction (XRD) pattern was measured on a Bruker-Axs D8 (GADDS) diffractometer using monochromated Cu Kα radiation. Atomic force microscopy (AFM) measurements were performed in air on a Veeco Dimension 3100 using a Nanosurf Easyscan 2 system fitted with a NCLR cantilever. Non-contact tapping mode was used to build a topological map of each samples over a 5 × 5 μm area and roughness statistics extracted using post-process software (Gwyddion).

### 4.4 Water bouncing tests

Water droplets were dropped from height of 7.5 cm using a 1 ml syringe without a dispensing tip to the CVD treated samples. The water droplets from this tip were 10 microliters in size. Methylene blue dye was added to the water to aid visualization; this did not change the behavior of the water droplets on the surface.

### 4.5 Bactericidal assay

A range of different samples (1 cm × 1 cm) were used in the antibacterial experiments: uncoated microscope glass (control), uncoated PDMS (PDMS), CVD treated PDMS (S-PDMS) and copper nanoparticle-encapsulated silicone (Cu-PDMS). These samples were evaluated against *Escherichia coli* ATCC 25922 and *Staphylococcus aureus* NCTC8325-4. The bacteria were stored at –70 °C in Brain–Heart-Infusion broth (BHI, Oxoid) containing 20% (v/v) glycerol and propagated on either MacConkey agar (MAC, Oxoid Ltd.) in the case of *E. coli* or Mannitol Salt agar (MSA, Oxoid Ltd.) in the case of *S. aureus*, for a maximum of 2 subcultures at intervals of 2 weeks.

BHI broth (10 ml) was inoculated with 1 bacterial colony and cultured in air at 37 °C for 17 h with shaking, at 200 rpm. The bacterial pellet was recovered by centrifugation (20 °C, 4000 × g, 5 min), washed in phosphate buffered saline (PBS, 10 ml) and centrifuged again (20 °C, 4000 × g, 5 min) to recover the bacteria, which were finally re-suspended in PBS (10 ml). The washed bacterial suspension was then diluted 1 in 1000 in PBS to give an inoculum of approximately 10^6^ cfu ml^–1^.

To ensure that the bacterial suspension did not bounce off the surface of the material, 10 μl of the inoculum was placed gently on to the material from a pipette tip held close to the surface and covered with a sterile cover slip (22 mm × 22 mm) to provide good contact between the bacteria and the surface of the sample. The samples were then incubated at room temperature for the allocated exposure time. Post irradiation, the inoculated samples and cover slips were placed into PBS in sterile plastic tubes and mixed on a vortex mixer for 20 seconds. The neat suspension and ten-fold serial dilutions were plated on the appropriate agar, incubated aerobically overnight at 37 °C and the colonies enumerated to determine the number of surviving bacteria. The bacterial numbers in the inocula were also determined in each experiment by viable colony counting. Each experiment included two technical replicates and the experiment was reproduced three times. The data was analyzed using the Mann–Whitney *U* test.

### 4.6 Bacterial attachment assay

Bacteria were grown from an overnight culture to 10^7^ cfu ml^–1^. Then, 10 μl of the bacterial suspension was placed on the specimen, which was placed at room temperature for 60 minutes. The specimens were then rinsed twice by gently dipping in PBS to remove any unattached cells. Afterwards, the samples were transferred into sterile conical tubes containing 3 ml of fresh PBS. The tubes were mixed using a vortex mixer for 5 minutes and then placed in an ultrasonic bath and sonicated for 15 minutes to release the attached cells from the biomaterial. After an additional vortex mix for 1 min, the suspensions were serially diluted with PBS and enumerated on the appropriate agar plates. The experiment was reproduced five times.

## Supplementary Material

Supplementary information

Supplementary movie
