# Supplementary material for: Copper-based water repellent and antibacterial coatings by aerosol assisted chemical vapour deposition
Source: Chem Sci. 2016 Apr 20;7(8):5126–31. doi: 10.1039/c6sc01150k (PMC6020121; doi:10.1039/c6sc01150k)
Supplement: Supplementary file 1 [file SC-007-C6SC01150K-s001.pdf]

Electronic Supplementary Material (ESI) for Chemical Science.
This journal is © The Royal Society of Chemistry 2016

Supplementary Information for

# Copper-based water repellent and antibacterial coatings by aerosol assisted chemical vapour deposition

Ekrem Ozkan,[a] Colin C. Crick,[b] Alaric Taylor,[c] Elaine Allan[d] and Ivan P. Parkin*[a]

[a]Materials Chemistry Research Centre, Department of Chemistry, University College London, 20 Gordon St, London, WC1H 0AJ, UK. Email: i.p.parkin@ucl.ac.uk, Tel: +44 (0)207 679 4669
[b]Department of Chemistry, Imperial College London, South Kensington Campus, SW7 2AZ, UK
[c]Department of Electronic and Electrical Engineering, University College London, Torrington Place, London, WC1E 7JE
[d]Division of Microbial Diseases, UCL Eastman Dental Institute, University College London, 256 Grays Inn Road, London, WC1X 8LD, UK

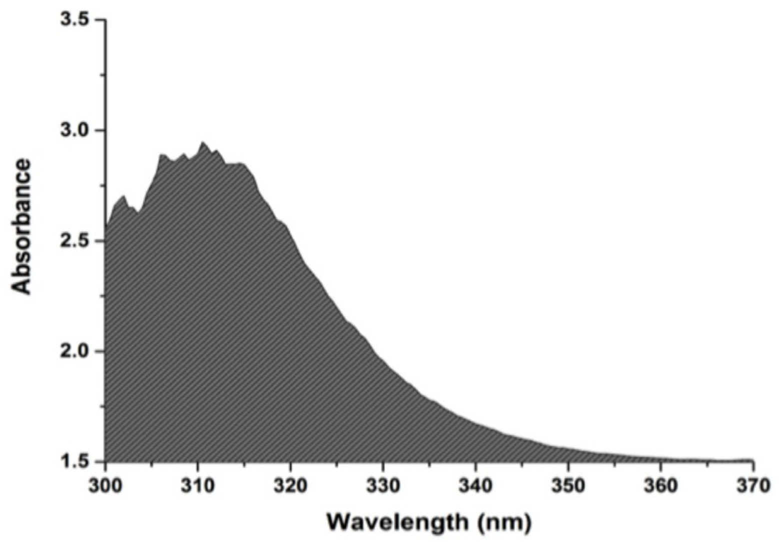


**Fig. S1.** UV-vis absorbance spectra of Cu-coated silicon polymer.

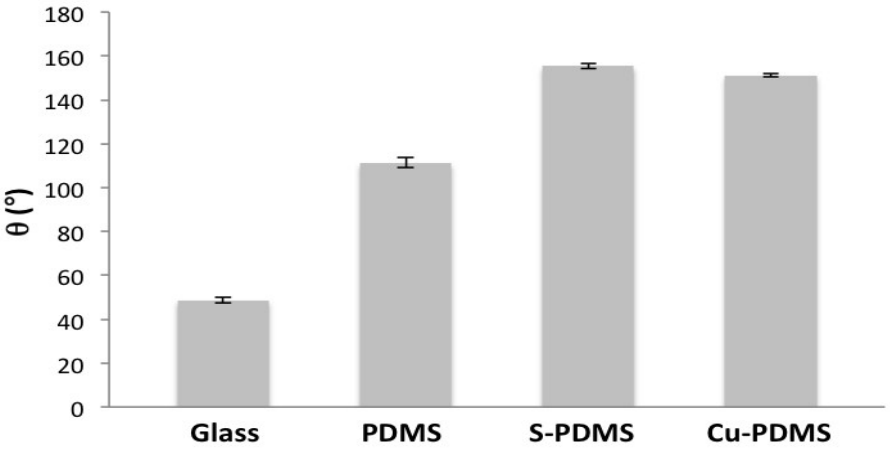


**Fig. S2.** Water contact angle of the samples. Error bars indicate standard deviations from five measurements.

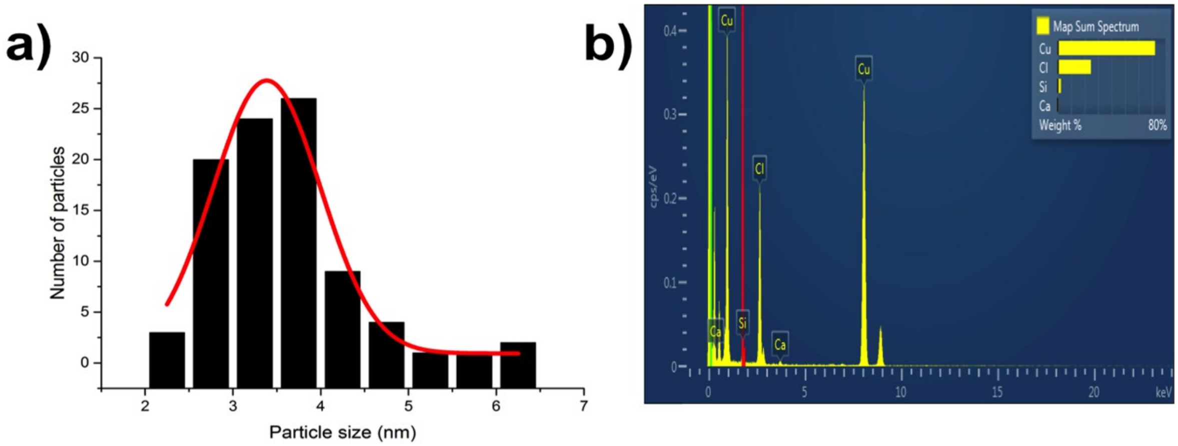


**Fig. S3.** (a) Particle size distribution histogram of colloidal Cu NPs. (b) energy dispersive X-ray spectrum of the Cu-PDMS composite.

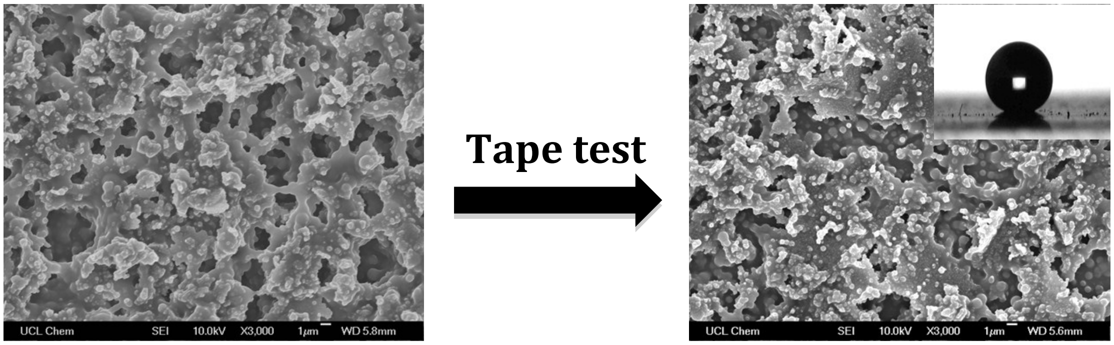


**Fig. S4.** SEM images of the Cu coated PDMS surface that was exposed to the tape test.
